# Q Fever: An Old but Still a Poorly Understood Disease

**DOI:** 10.1155/2012/131932

**Published:** 2012-11-19

**Authors:** Hamidreza Honarmand

**Affiliations:** Cellular and Molecular Research Center, Guilan University of Medical Sciences, Rasht, Iran

## Abstract

Q fever is a bacterial infection affecting mainly the lungs, liver, and heart. It is found around the world and is caused by the bacteria *Coxiella burnetii.* The bacteria affects sheep, goats, cattle, dogs, cats, birds, rodents, and ticks. Infected animals shed this bacteria in birth products, feces, milk, and urine. Humans usually get Q fever by breathing in contaminated droplets released by infected animals and drinking raw milk. People at highest risk for this infection are farmers, laboratory workers, sheep and dairy workers, and veterinarians. Chronic Q fever develops in people who have been infected for more than 6 months. It usually takes about 20 days after exposure to the bacteria for symptoms to occur. Most cases are mild, yet some severe cases have been reported. Symptoms of acute Q fever may include: chest pain with breathing, cough, fever, headache, jaundice, muscle pains, and shortness of breath. Symptoms of chronic Q fever may include chills, fatigue, night sweats, prolonged fever, and shortness of breath. Q fever is diagnosed with a blood antibody test. The main treatment for the disease is with antibiotics. For acute Q fever, doxycycline is recommended. For chronic Q fever, a combination of doxycycline and hydroxychloroquine is often used long term. Complications are cirrhosis, hepatitis, encephalitis, endocarditis, pericarditis, myocarditis, interstitial pulmonary fibrosis, meningitis, and pneumonia. People at risk should always: carefully dispose of animal products that may be infected, disinfect any contaminated areas, and thoroughly wash their hands. Pasteurizing milk can also help prevent Q fever.

## 1. Introduction

Q fever is a zoonosis caused by *Coxiella burnetii*, an obligate gram-negative intracellular bacterium. Most commonly reported in southern France and Australia, Q fever occurs worldwide. *C. burnetii* infects various hosts, including humans, ruminants (cattle, sheep, goats), and pets—and, in rare cases, reptiles, birds, and ticks. This bacterium is excreted in urine, milk, feces, and birth products. These products, especially the latter, contain large numbers of bacteria that become aerosolized after drying. *C. burnetii* is highly infectious, and only a few organisms can cause disease.

Because of its sporelike-life cycle, *C. burnetii* can remain viable and virulent for months. Infection can be acquired via inhalation or skin contact, and direct exposure to a ruminant is not necessary for infection. Rare human-to-human transmissions involving exposure to the placenta of an infected woman and blood transfusions have been reported [1]. Sexual transmission is also possible [2].

*C. burnetii* infection in livestock often goes unnoticed. In humans, acute *C. burnetii* infection is often asymptomatic or mistaken for an influenza-like illness or atypical pneumonia. In rare cases (<5%), *C. burnetii* infection becomes chronic [3], with devastating results, especially in patients with preexisting valvular heart disease. Because of its highly infectious nature and having an inhalational route of transmission, *C. burnetii* is recognized as a potential agent of bioterrorism. The Centers for Disease Control and Prevention (CDC) classify Q fever as a Category B agent.

The pathogenic agent is to be found everywhere except New Zealand [1]. The bacterium is extremely sustainable and virulent: a single organism is able to cause an infection. The common way of infection is inhalation of contaminated dust, contact with contaminated milk, meat, wool, and particularly birthing products. Ticks can transfer the pathogenic agent to other animals.

Some studies have shown more men to be affected than women [2, 3], which may be attributed to different employment rates in typical professions. “At risk” occupations include, but are not limited to: veterinary personnel, stockyard workers, farmers, shearers, animal transporters, laboratory workers handling potentially infected veterinary samples or visiting abattoirs, and people who cull and process kangaroos.

## 2. History

It was first described by Edward Holbrook Derrick [4] in abattoir workers in Brisbane, Queensland, Australia. The “Q” stands for “query” and was applied at a time when the causative agent was unknown; it was chosen over suggestions of “abattoir fever” and “Queensland rickettsial fever,” to avoid directing negative connotations at either the cattle industry or the state of Queensland [5].

The pathogen of Q fever was discovered in 1937, when Frank Macfarlane Burnet and Mavis Freeman isolated the bacterium from one of Derrick's patients [6]. It was originally identified as a species of *Rickettsia*. H.R. Cox and Gordon Davis isolated it from ticks in Montana, USA in 1938 [7]. *Coxiella burnetii* is no longer regarded as closely related to Rickettsiae, but as similar to *Legionella* and *Francisella*, and is a proteobacterium.

## 3. Bacteriology

*C. burnetii* is an obligate intracellular, small gram-negative bacterium (0.2 to 0.4 *μ*m wide, 0.4 to 1 *μ*m long). Although possessing a membrane similar to that of a gram-negative bacterium, it is usually not stainable by the Gram technique. The Gimenez method is usually used to stain *C. burnetii* in clinical specimens or laboratory cultures. Since *C. burnetii* cannot be grown in axenic medium and has long been recovered from ticks, it has been classified in the *Rickettsiales* order, the *Rickettsiaceae* family, and the *Rickettsiae* tribe together with the genera *Rickettsia* and *Rochalimaea* [8]. However, phylogenetic investigations, based mainly on 16S rRNA sequence analysis, have shown that the Coxiella genus belongs to the gamma subdivision of *Proteobacteria* [9], with the genera *Legionella*, *Francisella*, and *Rickettsiella* as its closest relatives.

*C. burnetii* expresses a low degree of genetic heterogeneity among strains by DNA-DNA hybridization. the genome size is highly variable among different *C. burnetii* strains, ranging from 1.5 to 2.4 Mb [10]. The inability to localize origin function by standard methods could well be related to the fact that *C. burnetii* probably has a linear rather than a circular chromosome and thus may not have conventional bidirectional replication [10]. *C. burnetii* gene sequences partially or completely available in the GenBank or EMBL databases include 23 chromosomal sequences and 17 plasmid sequences. The *C. burnetii* genome comprises facultatively a 36- to 42-kb plasmid, whose function remains undetermined.

*C. burnetii* displays antigenic variations similar to the smooth-rough variation in the family *Enterobacteriaceae*. Phase variation is related mainly to mutational variation in the lipopolysaccharide (LPS) [11]. Phase I is the natural phase found in infected animals, arthropods, or humans. It is highly infectious and corresponds to smooth LPS. In contrast, phase II is not very infectious and is obtained only in laboratories after serial passages in cell cultures or embryonated egg cultures. It corresponds to rough LPS. Compared to phase I, phase II displays a truncated LPS and lacks some protein cell surface determinants [12].

Genetic variability among different *C. burnetii* strains, as demonstrated by different RFLP-based genomic groups, specific plasmid regions, and LPS variations, were tentatively related to virulence. Genomic groups I, II, and III were associated with animal, tick, or acute Q fever human isolates, referred to as acute strains, whereas groups IV and V were associated with human Q fever endocarditis isolates, referred to as chronic strains. Group VI isolates, obtained from feral rodents in Dugway (Utah), were of unknown pathogenicity. Comparison of the various isolates for LPS variations, using sodium dodecyl sulfate-polyacrylamide gel electrophoresis and immunoblotting, resulted in isolates being placed into groups similar to the genomic groups [13]. Some investigations suggest that predisposing host factors are more important than genomic strain variation in the explanation of the occurrence of acute or chronic Q fever diseases in humans [14, 15]. Moreover, recent data shows that genetic variation has an apparently closer connection with the geographical source of the isolate than with the clinical presentation.

## 4. Clinical Manifestation

Clinical signs of Q fever are often subclinical or extremely mild. In acute infection. The incubation period has been estimated to be approximately 20 days (range 14 to 39 days). There is no typical form of acute Q fever. The clinical signs vary greatly from patient to patient. The most important diagnostic clue is the epidemiological circumstance. Typically, three major presentations are described. These are as follows.

- Self-limited flu-like syndrome. A self-limited flu-like syndrome is the most common manifestation of Q fever. In Spain, this form of Q fever has been demonstrated as the cause of 21% of episodes of fever lasting for more than 1 week and less than 3 weeks. The most frequent symptoms, usually following a sudden onset, are high-grade fever (104°F or 40°C), fatigue, headache, and myalgias. The duration of fever increases with increasing age.
- Pneumonia. Atypical pneumonia is one of the most commonly recognized forms of acute Q fever. Most cases are clinically asymptomatic or mild, characterized by a nonproductive cough, fever, and minimal auscultatory abnormalities, but some patients present with acute respiratory distress. Pleural effusion can also be present. Findings on the chest radiograph are nonspecific. Marrie et al. [16] demonstrated that 3.7% of all patients with community-acquired pneumonia admitted to a tertiary-care teaching hospital in Nova Scotia over a 5-year period were due to *C. burnetii*, which is similar to the findings of Lieberman et al. in Israel (5.8%) [17].
- Hepatitis (inflammation of the liver) is the predominant form of acute Q fever and it manifests mostly as a granulomatous hepatitis. The duration of symptoms varies from 10 to 90 days. The mortality rate ranges from 0.5 to 1.5%, depending upon the series [18].
- Hepatitis. Three major forms of hepatitis may be encountered: an infectious hepatitis-like form of hepatitis with hepatomegaly but seldom with jaundice, clinically asymptomatic hepatitis, and prolonged fever of unknown origin with characteristic granulomas on liver biopsy [19].
- Other manifestations. Many other clinical manifestations of acute Q fever are possible: maculopapular or purpuric exanthema in 10% of patients [18], pericarditis and/or myocarditis (which is frequently fatal), and severe headache. Aseptic meningitis and/or encephalitis, which occur in 0.2 to 1.3% of patients with Q fever [20], are rarely accompanied by seizures and coma. Polyradiculoneuritis, optic neuritis, hemophagocytosis, hemolytic anemia, transient hypoplastic anemia, thyroiditis, gastroenteritis, pancreatitis, lymphadenopathy mimicking lymphoma, erythema nodosum, bone marrow necrosis, inappropriate secretion of antidiuretic hormone, mesangioproliferative glomerulonephritis related to antiphospholipid antibodies, and splenic rupture are uncommon manifestations of acute Q fever [21].

Chronic infection: chronic Q fever was initially described as lasting for more than 6 months after the onset. It occurs in approximately 5% of patients infected with *C. burnetii* and may develop insidiously months to years after the acute disease. In the chronic form of Q fever, *C. burnetii* multiplies in macrophages, and a permanent rickettsemia results in very high levels of persistent antibodies. Typically, the heart is the most commonly involved organ, followed by arteries, bones, and liver [22]. Endocarditis usually occurs in patients with previous valvular damage or those who are immunocompromised [23]. Chronic Q fever represents 3% of all cases of endocarditis in England and Lyon, France [24], and 15% in Marseille, France [25], and its annual incidence is 0.75 cases per 1 million population in Israel [26]. Clinically, the disease usually presents as a subacute or acute blood culture-negative endocarditis [27]. Symptoms are not specific. Arterial embolism occurs in about 20% of patients [28]. Vegetations are only rarely seen by transthoracic cardiac ultrasonography. They are usually smooth and nodular [27]. Because of the lack of specificity of symptoms, the diagnosis is often delayed 12 to 24 months, resulting in an increased mortality rate. Other manifestations of chronic Q fever include infections of aneurysms or vascular grafts [25], isolated hepatitis possibly complicated by hepatic fibrosis and cirrhosis [22], and osteoarthritis and osteomyelitis [29]. Rare cases of pericardial effusion [30], pulmonary interstitial fibrosis, pseudotumor of the lung, lymphoma-like presentation, amyloidosis, and mixed cryoglobulinemia have been reported in the literature.

Q fever during pregnancy. Both acute and chronic Q fever have been described during pregnancy. In mammals, *C. burnetii* undergoes reactivation during pregnancy and thus is responsible for higher rates of abortion, prematurity, and low birth weight [31]. In humans, it has been isolated from the placenta of a woman who became pregnant 2 years after an episode of acute Q fever [32], but few cases have been reported. Clinically, although most cases seem to be asymptomatic [33], complications may complicate the course of the disease, such as in utero fetal death [34], placentitis, or thrombocytopenia [35]. Although intrauterine transmission of *C. burnetii* has been documented, the consequences of congenital Q fever remain to be determined.

## 5. Epidemiology

Q fever is a worldwide zoonosis. The reservoirs are extensive but only partially known and include mammals, birds, and arthropods, mainly ticks. While an important reservoir seems to be small wild rodents, the most commonly identified sources of human infection are farm animals such as cattle, goats, and sheep. Pets, including cats [36], rabbits, and dogs, have also been demonstrated to be potential sources of urban outbreaks. Cats are suspected as an important reservoir of *C. burnetii* in urban areas and may be the source of urban outbreaks [37–39]. In Canada, 6 to 20% of cats have anti-*C. burnetii* antibodies [36]. Wild rats have been suspected as an important reservoir in Great Britain [40]. All these mammals, when infected, shed the desiccation-resistant organisms in urine, feces, milk, and, especially, birth products [41]. Reactivation of infection occurs in female mammals during pregnancy. Q fever causes abortions in goats and, less frequently, sheep and causes reproductive problems in cattle [42]. High concentrations of *C. burnetii* (up to 10^9^ bacteria per g of tissue) are found in the placentas of infected animals. Due to its resistance to physical agents, probably related to its sporulation process [43], *C. burnetii* survives for long periods in the environment.

In humans, infection results from inhalation of contaminated aerosols from amniotic fluid or placenta or contaminated wool. Therefore, Q fever is an occupational hazard. At greatest risk are persons in contact with farm animals, but also at risk are laboratory personnel who work with infected animals. When looking for the source of *C. burnetii* exposure, the investigator should search for contact with a parturient or newborn animal. Mammals also shed *C. burnetii* in milk, and thus, consumption of raw milk could be a source of infection [44]. Sexual transmission of Q fever has been demonstrated in the mouse [45] and has been suspected in humans [46]. Sporadic cases of human-to-human transmission following contact with an infected parturient woman have been reported and have been suspected to occur by direct aerosol transmission. It has also been proven to occur via transplacental transmission, resulting in congenital infections [34], via intradermal inoculation, and via blood transfusion [47]. Ticks transmit *C. burnetii* to domestic mammals but not to humans [41]. *C. burnetii* may persist asymptomatically in humans throughout life. However, pregnancy, a cardiac valvular abnormality, a vascular aneurysm or prosthesis, hemodialysis [48], and immunodeficiency, including AIDS [49], may promote reactivation of dormant *C. burnetii*.

In Europe, acute Q fever cases are more frequently reported in spring and early summer. They may occur at all ages, but they are more frequent in men than in women. Q fever is usually benign, but mortality occurs in 1 to 11% of patients with chronic Q fever [50]. *C. burnetii* is endemic in every part of the world except New Zealand [51]. Since the clinical presentation is very pleomorphic and nonspecific, the incidence of Q fever among humans is probably underestimated, and diagnosis particularly relies upon the physician's awareness of the symptoms of Q fever and the presence of a reliable diagnostic laboratory. In southern France, 5 to 8% of cases of endocarditis are due to *C. burnetii*, and the prevalence of acute Q fever is 50 cases per 100,000 inhabitants [18]. Seroepidemiological surveys have shown that 18.3% of blood donors in Morocco, 26% in Tunisia [52], 37% in Zimbabwe [53], 44% in Nigeria [54], 10 to 37% in northeast Africa, and 14.6 to 36.6% in different areas of Canada [22] had anti-*C. burnetii* antibodies. Large outbreaks of Q fever have also been reported in the Basque country in Spain [55], in Switzerland [56], in Great Britain [57], and in Berlin, Germany [58].

In addition, a large number of Q fever cases have been reported in The Netherlands since 2007, with over 3700 human cases reported through March 2010. Infected dairy goat farms are believed to be the source of the outbreak, and most human cases have been reported in the southern region of the country [59].

## 6. Laboratory Diagnosis

Blood cultures are typically negative (Note that, although possible, attempting to isolate the organism from blood is a dangerous practice; cases of Q fever have developed in laboratory technicians).

*C. burnetii* can be seen on smears or frozen tissue prepared with a routine Giemsa stain. Histopathologic changes consistent with doughnut granulomata the liver and bone marrow may be observed, but these are not specific for *C. burnetii*. They can also occur in Hodgkin lymphoma, typhoid fever, cytomegalovirus infection, infectious mononucleosis, and allopurinol hypersensitivity.

Most cases of Q fever are diagnosed based on detection of phase I and II antibodies (between acute and convalescent paired sera); a 4-fold rise in complement-fixing antibody titer against phase II antigen occurs and yields the highest specificity. This requires a baseline sample and another sample in 3-4 weeks. Thus, serologic tests are not helpful acutely but may later confirm the diagnosis: seroconversion generally occurs between days 7 and 15 and is almost always present by 21 days.

The 3 serologic techniques used for diagnosis include indirect immunofluorescence (IIF) (method of choice), complement fixation, and enzyme-linked immunosorbent assay (ELISA) (comparable to IIF). As noted above, significant titers may take 2–4 weeks to appear. Laboratory values vary considerably, so clinicians must interpret results according to their local standards [60].

Raoult et al. recommended serologic testing 2 years following treatment in patients with valvulopathy after acute infection [61], whereas Healy et al. recommended serial testing every 4 months for 2 years, with additional investigation in those with elevated phase 1 immunoglobulin G (IgG) titers greater than 800 [62]. Maurin and Raoult recommendations are shown in Table 1 [63].

Serologic followup to detect a rise in phase I IgG titers of 1 : 800 or more can be performed twice every 3 months. If detected, transesophageal echocardiography and serum real-time polymerase chain reaction (PCR) techniques can be performed in an attempt to diagnose endocarditis [61, 63]. Sensitivities may be as low as 18% in early disease. 

Interpretation of Q fever serology is challenging in regard to discordance of the serologic results from different reference laboratories [64]. None of these results should be used in isolation, and their interpretation should always be applied in the appropriate clinical context. False-positive serologic results may occur in legionellosis and leptospirosis. 

IIF findings in acute Q fever include the following.

- A rise in IgG and IgM against phase II antigen [65].
- Phase II IgM of 1 : 50 or more; usually undetectable after 4 months but can last 12 months or more.
- Phase II IgG of 1 : 200 or more.
- Phase II titers of 1 : 100 or less make the diagnosis of acute Q fever unlikely.
- In a reference French laboratory, these values showed 100% specificity.

IIF findings in chronic Q fever include the following.

- A rise in IgG and IgA against phase I antigen [65, 66].
- Phase I IgG of 1 : 800 or more is considered diagnostic of endocarditis (one of major modified Duke criteria).
- Phase II IgM titers are lower or absent.
- Phase II IgG titers are usually greater than 1 : 1600; they can last up to 12 years after an outbreak.
- The main predictive criterion of clinical cure is detection of phase I IgG titer of less than 1 : 200.

Complement fixation is less sensitive and specific than IIF, and the time to positivity may take longer than IIF. Different cutoff values are also used. IgG levels usually fall within 3 years. 

In acute Q fever, the anti-IgG titer is at least 200, and the anti-IgM titer level is at least 50. In chronic Q fever, the anti-IgA titer for phase I is greater than 50, and the anti-IgG titer for phase I is greater than 800.

In certain reference laboratories, polymerase chain reaction (PCR) techniques can be used with tissue specimens, such as resected cardiac valves, with greater sensitivity than serum assays, but these are not generally available commercially. *C. burnetii* organisms can persist in tissues even after prolonged antimicrobial treatment [66]. Although still controversial, serum PCR may be used to diagnose acute Q fever in the first 2 weeks of the disease. It should also be reserved for seronegative patients in the subsequent 2 weeks and not used later than 4 weeks following onset [62].

## 7. Prevention

In the United States, Q fever outbreaks have resulted mainly from occupational exposure involving veterinarians, meat processing plant workers, sheep and dairy workers, livestock farmers, and researchers at facilities housing sheep. Prevention and control efforts should be directed primarily toward these groups and environments.

The following measures should be used in the prevention and control of Q fever.

- Educate the public on sources of infection.
- Appropriately dispose of placenta, birth products, fetal membranes, and aborted fetuses at facilities housing sheep and goats.
- Restrict access to barns and laboratories used in housing potentially infected animals.
- Use only pasteurized milk and milk products.
- Use appropriate procedures for bagging, autoclaving, and washing of laboratory clothing.
- Vaccinate (where possible) individuals engaged in research with pregnant sheep or live *C. burnetii*.
- Quarantine imported animals.
- Ensure that holding facilities for sheep should be located away from populated areas. Animals should be routinely tested for antibodies to *C. burnetii*, and measures should be implemented to prevent airflow to other occupied areas.
- Counsel persons at highest risk for developing chronic Q fever, especially persons with preexisting cardiac valvular disease or individuals with vascular grafts.

A vaccine for Q fever has been developed and has successfully protected humans in occupational settings in Australia. However, this vaccine is not commercially available in the United States. Persons wishing to be vaccinated should first have a skin test to determine a history of previous exposure. Individuals who have previously been exposed to *C. burnetii* should not receive the vaccine because severe reactions, localized to the area of the injected vaccine, may occur. A vaccine for use in animals has also been developed, but it is not available in the United States.

## 8. Treatment

Doxycycline is the first line treatment for all adults, and for children with severe illness. Treatment should be initiated immediately whenever Q fever is suspected. Use of antibiotics other than doxycycline or other tetracyclines is associated with a higher risk of severe illness. Doxycycline is most effective at preventing severe complications from developing if it is started early in the course of disease. Therefore, treatment must be based on clinical suspicion alone and should always begin before laboratory results return. If the patient is treated within the first 3 days of the disease, fever generally subsides within 72 hours. In fact, failure to respond to doxycycline suggests that the patient's condition might not be due to Q fever. Severely ill patients may require longer periods before their fever resolves. Resistance to doxcycline has not been documented [3].

There is no role for prophylactic antimicrobial agents in preventing Q fever after a known exposure and prior to symptom onset; attempts at prophylaxis will likely extend the incubation period by several days but will not prevent infection from occurring. Patients should be treated for at least 3 days after the fever subsides and until there is evidence of clinical improvement. Standard duration of treatment is 2-3 weeks [3].

The use of doxycycline is recommended to treat Q fever in children of all ages who are hospitalized or are severely ill. Unlike older generations of tetracyclines, doxycycline has not been shown to cause staining of permanent teeth, and most experts consider the benefit of doxycycline in treating Q fever in children younger than 8 years of age with severe illness or who are hospitalized greater than the potential risk of dental staining. Children with mild illness who are less than 8 years of age may be treated with co-trimoxazole, but therapy should be switched to doxycycline if their course of illness worsens.

In cases of life-threatening allergies to doxycycline and in pregnant patients, physicians may need to consider alternate antibiotics. Treatment of pregnant women diagnosed with acute Q fever with once daily co-trimoxazole throughout pregnancy has been shown to significantly decrease the risk of adverse consequences for the fetus [3, 67].

Recommended treatment for Chronic Q fever in Adults is: Doxycycline 100 mg every 12 hours and hydroxychloroquine 200 mg every 8 hours. Standard duration of treatment is 18 months [3].

## 9. Conclusion

Although described 60 years ago, Q fever is still a poorly understood disease. Its reservoirs seem to be related to any mammal, but ticks may also be reservoirs. The clinical presentation is very pleomorphic and includes severe forms with a poor prognosis. Most often, acute cases present as asymptomatic infections, as a flu-like syndrome, as a pneumonia, or as hepatitis. Host factors probably play an important role in the development of chronic disease, which may present as a blood-culture-negative endocarditis or as an infected aneurysm. Although its exact prevalence is unknown, it is likely that the number of cases of Q fever is underestimated. Therefore, the diagnosis must be considered in the case of an unexplained fever, especially if the fever recurred following contact with possibly contaminated mammals. The best tests for diagnosis are those which permit the direct detection of bacteria. They include shell vial cell culture, PCR amplification, and immunodetection with tissue biopsy specimens. All these techniques require a level 3 biosafety laboratory and trained personnel due to the extreme infectivity of *C. burnetii*. In chronic cases, the techniques that allow the direct detection of *C. burnetii* in blood or tissues should be used before the beginning of therapy. As for indirect specific diagnosis, the technique to be used should be very sensitive and should detect antibodies early in the course of the disease. Although many techniques have been described, immunofluorescence assay is the reference method. It is both very specific and sensitive. In case of acute Q fever, diagnosis would be confirmed by an immunofluorescence assay titer greater than or equal to the cutoff value (which must be determined for each geographical area) or by a fourfold increase in the antibody titer detected by immunofluorescence assay, complement fixation, ELISA, or microagglutination. The presence of cross-reacting antibodies should be investigated by cross-adsorption followed by Western immunoblotting.

## Figures and Tables

**Table 1:** Cutoff proposal for Q fever diagnosis by using microimmunofluorescence and interpretation of serological results obtained with a single serum sample.

Phase II antibody titer		Phase I antibody titer (IgG)	Interpretation
IgG	IgM		
≤100			Active Q fever improbable
≥200	≥50		Acute Q fever (100% predictive)
		≥1 : 800	Chronic Q fever (98% predictive)
		≥1 : 1,600	Chronic Q fever (100% predictive)
